# COVID-19 and (mis)understanding public attitudes to social security: Re-setting debate

**DOI:** 10.1177/02610183221091553

**Published:** 2023-02

**Authors:** Michael Orton, Sudipa Sarkar

**Affiliations:** 2707 University of Warwick, England; 2707 University of Warwick, England

**Keywords:** public attitudes, social security, welfare benefits

## Abstract

The Covid-19 pandemic has seen emerging debate about a possible shift in ‘anti-welfare commonsense’ i.e. the orthodoxy previously described in this journal as solidifying negative public attitudes towards ‘welfare’. While a shift in attitudes might be ascribed to the circumstances of the crisis it would still be remarkable for such a strongly established orthodoxy to have changed quite so rapidly. It is appropriate, therefore, to reflect on whether the ‘anti-welfare’ orthodoxy was in fact as unequivocal as claimed? To address this question, challenges to the established orthodoxy that were emerging pre-pandemic are examined along with the most recently available survey data. This leads to discussion of broader issues relating to understanding attitudes: methodology; ‘messiness’ and ambivalence of attitudes; attitudes and constructions of deservingness; and following or leading opinion. It is argued that the ‘anti-welfare’ orthodoxy has always been far more equivocal than claimed, with consequent implications for anti-poverty action and re-setting debate.

## Introduction

The concern of this article is the argument that there is a commonly held view that the majority of the public in Britain regard ‘welfare’^1^ as inherently and inevitably negative and problematic (Hudson et al. 2016a: 215). This negative characterisation can be seen in what Jensen (2014) calls the ‘machine of welfare common sense’, a powerful combination of political narrative and popular media rhetoric. Examples range from the language of strivers versus skivers to the ‘poverty porn’ of television programmes such as *Benefits Street* (ibid.) and with the scapegoating of benefit claimants a particular feature. The intensity of the ‘machine of welfare commonsense’ has been cogently demonstrated in *Critical Social Policy* by Jensen and Tyler’s (2015) consideration of a particular case relating to larger families.^2^ But there is also a longevity with pervasive, negative depictions of ‘welfare’ and its recipients having over time become a staple of political discourse and media stories.

Jensen and Tyler (2015: 470) conclude that there is an excellent evidence-base around solidifying negative public attitudes towards ‘welfare’. The position is summarised by Deeming and Johnston (2018: 400), in an article entitled ‘Coming together in a rightward direction’, as being that since the mid-1990s there has been a strong trend in British attitudes away from a ‘pro-welfare’ stance towards a neo-liberal, ‘workfare not welfare’, position and “Society has converged on anti-welfare attitudes” (ibid: 412). The ‘anti-welfare’ thesis is comprehensive, unequivocal and, as Geiger and Meueleman (2016) and Hudson et al. (2016a) note, it has become an established orthodoxy.

In policy terms, ‘anti-welfare commonsense’ can be seen in the ‘welfare reform’ agenda that began in 2010 with the Conservative-Liberal Democrat Coalition Government. It has continued with Conservative governments since 2015, albeit many of the developments having roots in preceding decades (for example see Dwyer and Wright, 2014; Finch and Gardiner, 2018; Millar and Bennett, 2017). Policy is exemplified by the eponymous Welfare Reform Act 2012 and the introduction of Universal Credit, alongside other measures creating an increasingly punitive and harsh system marked by sanctions and conditionality. Homelessness and food banks are just two of the most visible signs of the inadequacy of provision (Lister, 2016). Criticisms have been powerfully articulated by the United Nations Special Rapporteur on extreme poverty and human rights, Philip Alston, who having visited Britain reflected that for almost one in every two children to be poor in twenty-first century Britain is “not just a disgrace, but a social calamity and an economic disaster, all rolled into one” (United Nations Human Rights – Office of the High Commissioner, 2018).

However, the Covid-19 crisis has included potential challenges to the established ‘anti-welfare commonsense’. The initial economic impact of the pandemic included the number of people in receipt of Universal Credit surging from 3 million in March 2020 to 5.2 million by May 2020 and continuing to increase to reach a figure of 5.8 million at the end of the year (Mackley, 2021). Between 16 and 31 March 2020 alone, 950,000 million people applied successfully for Universal Credit which is ten times the usual number of claimants in any given fortnight. In addition to managing this new level of claims, some changes were made to make benefits more generous (or perhaps more accurately, less parsimonious). For example, the basic allowances in Universal Credit and Working Tax Credit were increased by £20 per week; the Universal Credit Minimum Income Floor for self-employed people was suspended; (contributory) Employment and Support Allowance was made payable from the first day of a claim not the eighth as was the case; and Local Housing Allowance (which provides support for rent) was raised (see Machin, 2021; Simpson, 2020).

More broadly, the benefits system has become the subject of considerable debate. The start of the pandemic saw attention being drawn to the low level of benefit rates e.g., Statutory Sick Pay being £94 a week. Headlines appeared such as ‘Pandemic poverty: “I can't afford to live on £94 a week”’ and ‘Coronavirus: How is £94 a week going to pay anyone’s bills?’ An initiative by footballer Marcus Rashford around free school meals and child poverty attracted considerable attention and support.^3^ In 2021 anti-poverty organisations such as the Joseph Rowntree Foundation launched a campaign to retain the £20 increase in Universal Credit. That campaign was unsuccessful but the government did alter the Universal Credit taper rate, meaning claimants in employment can retain a larger proportion of their earnings. Deville and Florisson (2021) argue that continuing economic difficulties mean government will face ongoing pressure to engage in wholesale reform of the social security system.

Another element of debate has been about the possibility of a shift in ‘anti-welfare commonsense’. For example, Hannah (2020) argues that with large numbers of households becoming reliant on government support, low-paid workers being recognised as key to the nation's wellbeing and attention being drawn to the reality of the inadequacy of benefit rates, the crisis might change the dominant UK narrative on ‘welfare’. Ryan (2020) makes a similar point, contending that the crisis has drawn attention to the failings of the benefits system meaning previous public indifference to cuts in social security may change and make it more difficult for government to undertake further cuts in future. Hinsliff (2021) notes that understanding benefits do not cover living costs in a pandemic can easily grow into a realisation that they are not enough in normal times either. Debate about a possible shift in ‘anti-welfare commonsense’ is currently driven by media commentators and it will be some years before survey data will enable empirical examination of the impact of the pandemic on public attitudes. However, it is evident that the positive changes to benefits noted above were introduced without controversy and campaigns such as by Marcus Rashford have not been met with derision, despite both being at odds with the ‘anti-welfare’ orthodoxy. This possible shift is reinforced by a finding that 62 per cent of people were in favour of retaining the £20 increase in Universal Credit (Proctor, 2021).

Even in the circumstances of the pandemic it seems remarkable, however, that such a strongly established orthodoxy could have changed quite so rapidly and hence the question arises of whether the ‘anti-welfare’ orthodoxy was perhaps as unequivocal as claimed? The article addresses that question and is in three parts. It begins by examining survey data. This includes using the most recently available data and sources beyond those typically drawn upon in the literature. Second, consideration is given to broader issues relevant to understanding ‘welfare attitudes’: methodology; ‘messiness’ and ambivalence of attitudes; attitudes and constructions of deservingness; and following or leading opinion. Third is discussion of the implications of the research findings for a number of debates and, with concern about the hardening attitudes thesis having primarily come from anti-poverty actors, particular attention is given to the theme of re-setting debate in relation to anti-poverty action.

### Survey data on public attitudes to ‘welfare’

Pre-pandemic the ‘anti-welfare’ orthodoxy was dominant, but some counter arguments were emerging. For example, Hudson et al. (2016b) question whether there was a truly golden age of positive attitudes while Clery (2016) notes some survey findings do not accord with the hardening attitudes thesis. More specifically, Geiger and Meueleman (2016: 292) argue the orthodoxy that public attitudes have become more hostile on ‘welfare’ can blind us to the nuances of shifts in public opinion and that while there is some evidence that attitudes to the benefits system have hardened, the scale and uniformity of these shifts is *perceived* to be greater than the evidence bears out.

The starting point is therefore to examine data on public attitudes. This section does so, beginning with the ‘anti-welfare’ argument.

### Evidence of ‘anti-welfare’ attitudes

Deeming and Johnston (2018) can be used as an exemplar of the ‘anti-welfare’ orthodoxy. It was noted above that the authors argue public opinion has hardened, moving in a rightward direction towards a neo-liberal ‘workfare not welfare’ position and converging on ‘anti-welfare attitudes’. The orthodoxy holds there is a clear, inexorable and unambiguous hardening of opinion. Deeming and Johnston’s study (ibid.) is based on analysis of responses to three questions in the British Social Attitudes survey (hereafter, BSA).^4^ The three questions are: ‘Around here most people could find a job if they really wanted one’; ‘Welfare benefits are either too low and cause hardship or too high and discourage work’; and, ‘If benefits weren’t so generous people would learn to stand on their own two feet’.

**Figure 1.**
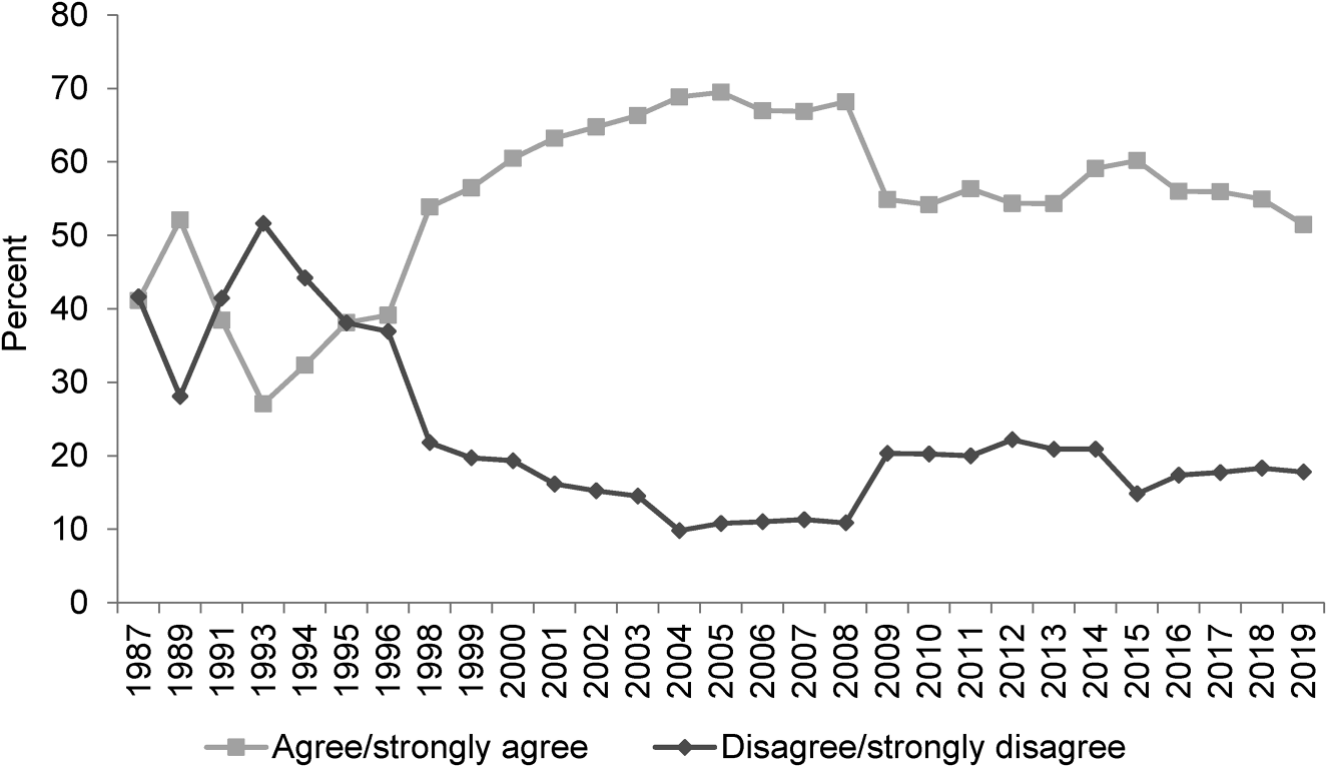
Around here most people could find a job if they really wanted one. Source: British Social Attitudes Survey, 1987 to 2019.

**Figure 2.**
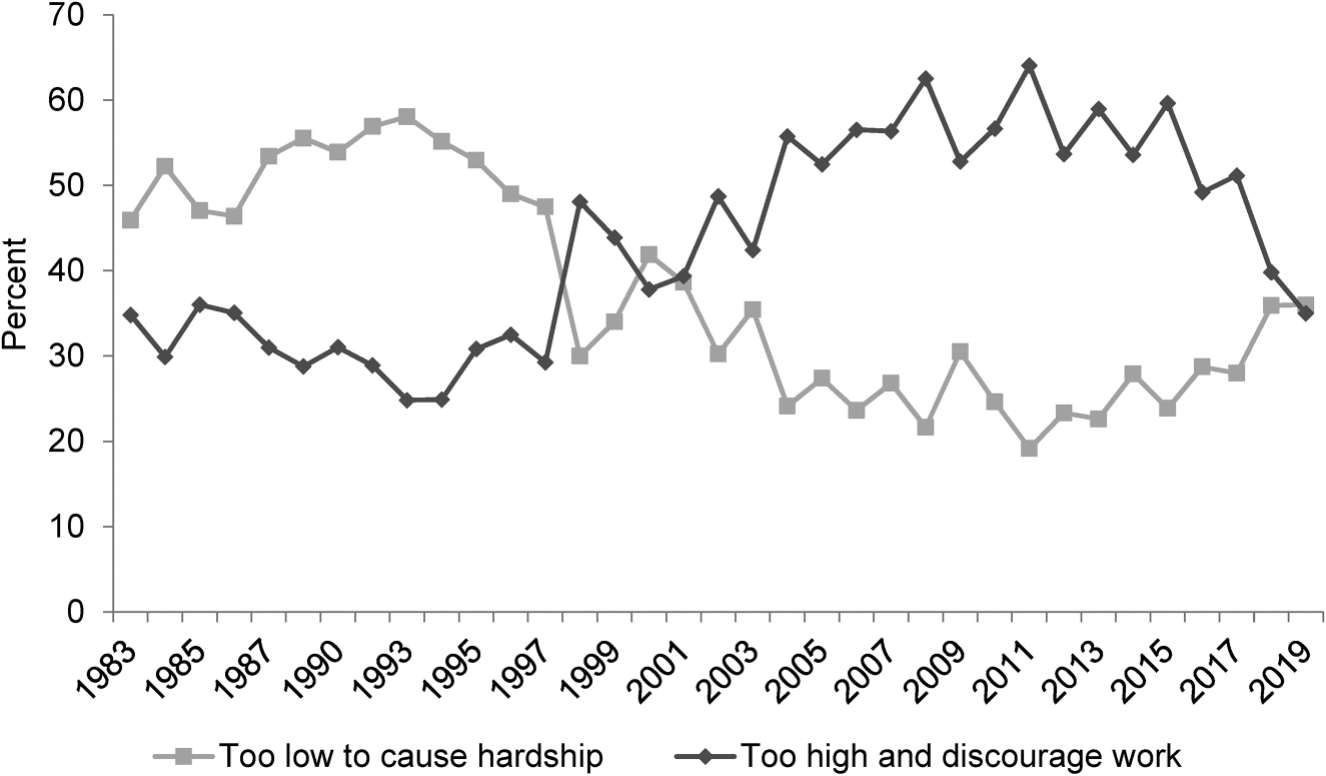
Welfare benefits are either too low and cause hardship or too high and discourage work. Source: British Social Attitudes Survey, 1983 to 2019.

**Figure 3.**
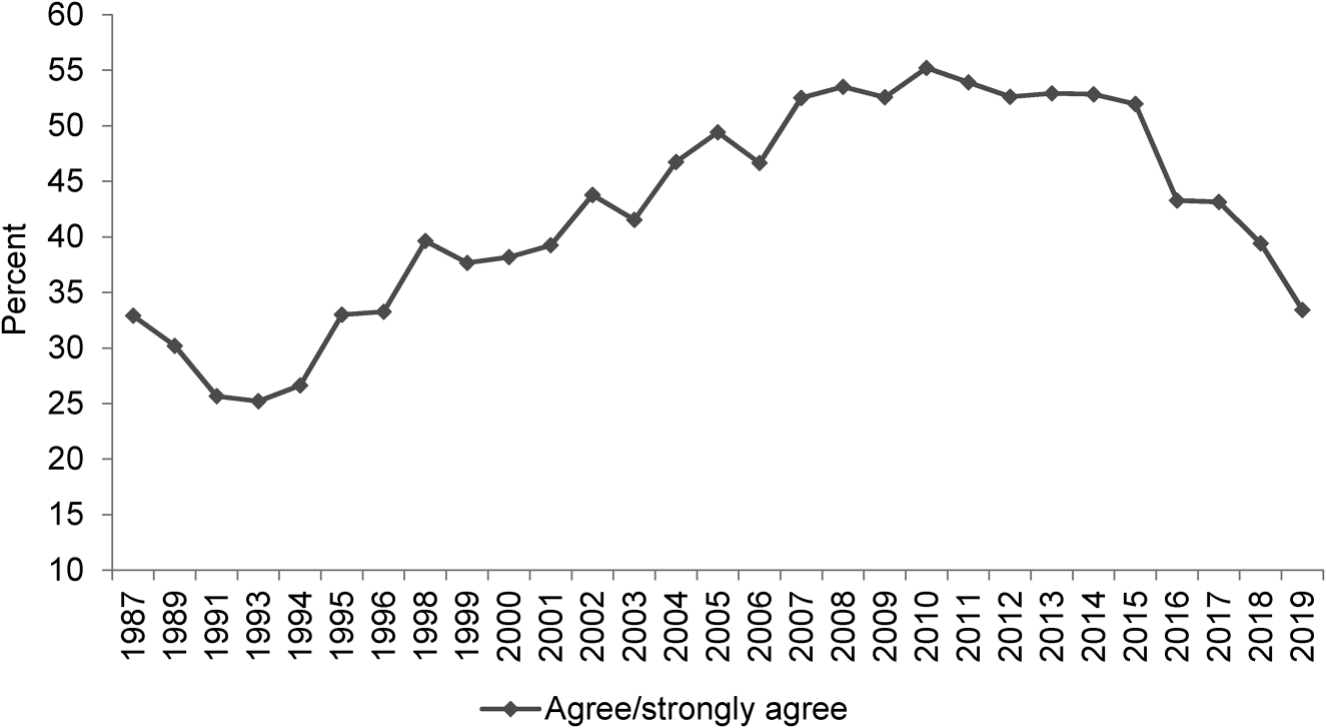
If benefits weren’t so generous people would learn to stand on their own two feet. Source: British Social Attitudes Survey, 1987 to 2019.

Figures 1, 2 and 3 show findings from these three questions. Deeming and Johnston’s analysis went up to 2012 but these Figures include the most recently available data i.e. through to 2019, dependent upon when each question was last asked.

Beginning with ‘Around here most people could find a job if they really wanted one’, it is clear from Figure 1 that in response to this BSA question there has been a hardening of attitudes. From a starting point in 1987 of an equal split between people agreeing and disagreeing with the proposition (both at 40 per cent), there was fluctuation through to 1996 but then significant divergence with the proportion of people agreeing with the statement rising to close to 70 per cent by the midpoint of the first decade of the twenty first century and disagreement falling to a low point of around 10 per cent. Since 2008 there has been some softening but nowhere near enough to counter earlier changes. Agreement with the proposition has always remained above 50 per cent while disagreement has at best reached a little over 20 per cent, with that being the case in 2019 which is the most recent year for which data on this question are available.

On the next two BSA questions to be considered (‘Welfare benefits are too high and discourage work’ and ‘If benefits weren’t so generous people would learn to stand on their own two feet’) findings, however, are not so straight-forward. As already noted, Deeming and Johnston’s (2018) analysis used data up to 2012 and in Figures 2 and 3 it is evident that attitudes had at that point hardened significantly since the 1980s. In both cases the percentage of people agreeing with these negative propositions increased from just over 30 per cent in the 1980s to over 50 per cent in 2012.

However, data since around 2015 show a considerable softening of attitudes. In 2019 the ‘benefits are too high and discourage work’ option was supported by only 35 per cent of respondents, slightly lower than the 36 per cent of people disagreeing. Responses on this question are still harder now than in 1983 but the inexorable rightward direction of attitudes is no longer the case. The question, ‘If benefits weren’t so generous people would learn to stand on their own two feet’, shows an even more dramatic shift. Since 2015 the percentage agreeing has fallen from close to 60 per cent to just 35 per cent in 2019. Comparing responses to the question in 1987 and 2019, contemporary attitudes are only a single percentage point more negative.

**Figure 4.**
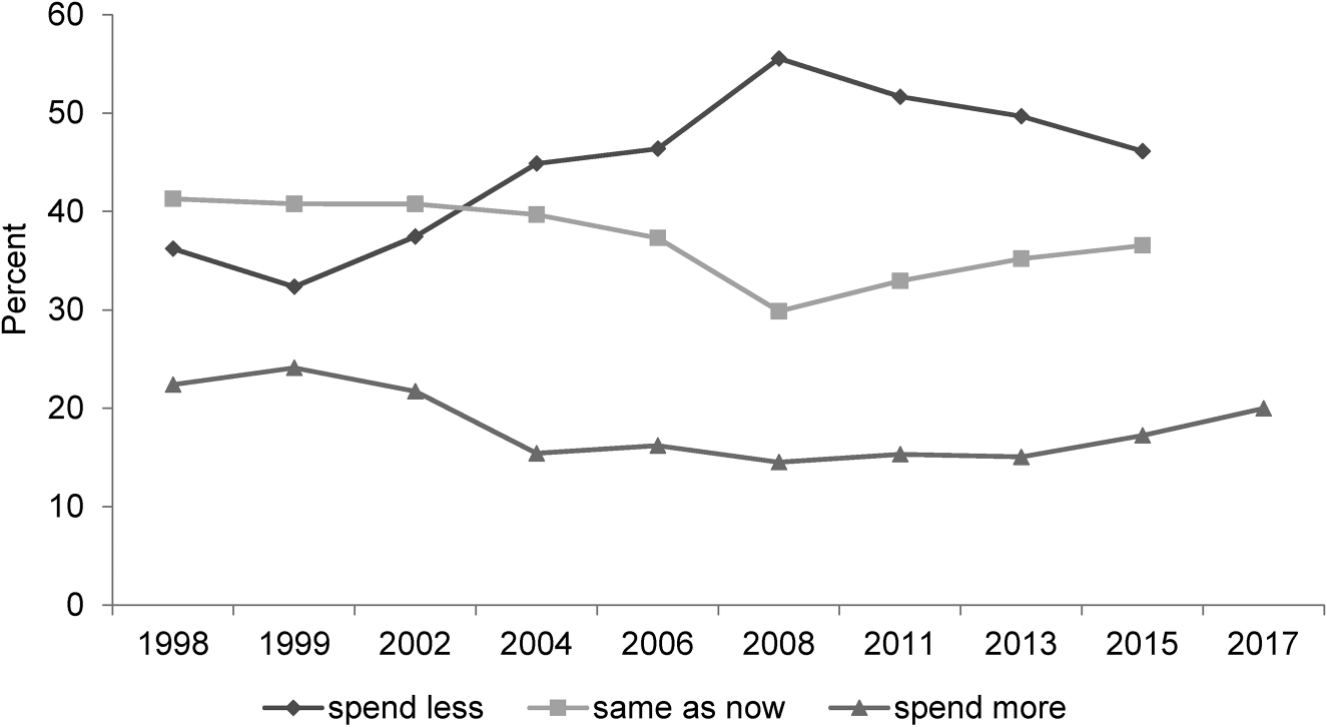
Would you like to see more or less government spending than now on benefits for the unemployed? Source: British Social Attitudes Survey, 1998 to 2016; the 2017 percentage is from Phillips et al. (2018).

**Figure 5.**
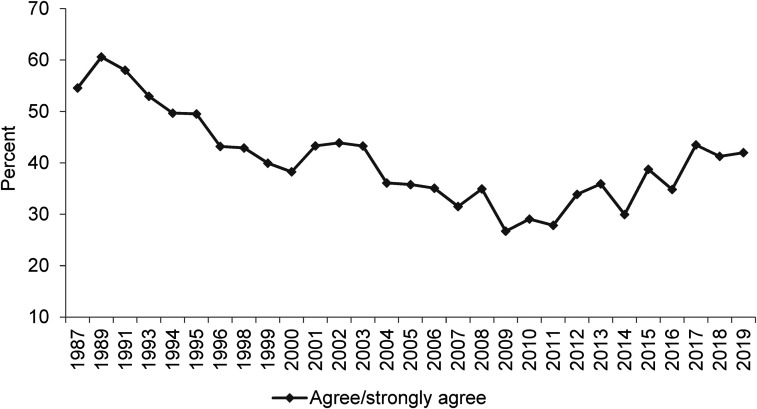
Government should spend more money on welfare benefits for the poor, even if it leads to higher taxes. Source: British Social Attitudes Survey, 1987 to 2019.

While not used by Deeming and Johnston (2018), two other BSA questions are often drawn upon in support of the ‘anti-welfare’ orthodoxy. These are: ‘Would you like to see more or less government spending than now on benefits for the unemployed’ and ‘Government should spend more money on welfare benefits for the poor, even if it leads to higher taxes’. Results from these two questions are presented in Figures 4 and 5.

Figures 4 and 5 show responses are not as clear cut as the ‘anti-welfare’ orthodoxy suggests they might be. Both Figures show that attitudes in the most recent BSA surveys in which the questions were asked, are harder than when first asked in the 1980s/1990s. That provides support for the ‘anti-welfare’ orthodoxy. But the Figures also show that since 2008 there has been a softening of attitudes in response to both questions. As in Figure 1, this softening is not enough to counter earlier change but it does not conform with an ongoing solidifying of ‘anti-welfare’ attitudes. This point will now be discussed further.

### A more equivocal picture

**Figure 6.**
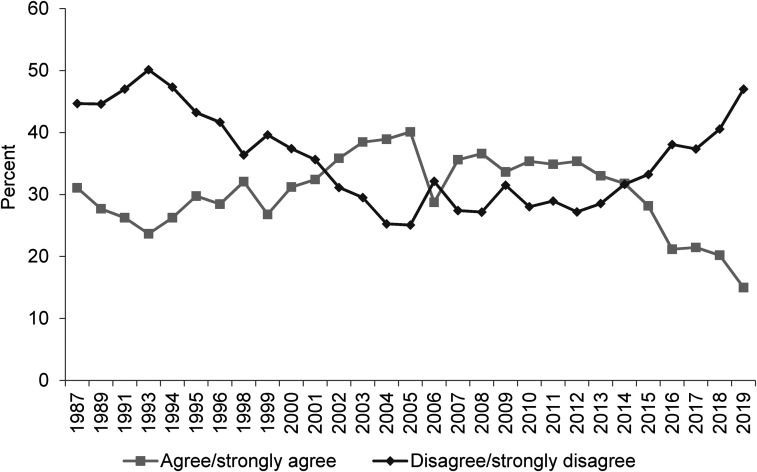
Many people who get social security don’t really deserve any help. Source: British Social Attitudes Survey, 1987 to 2019.

**Figure 7.**
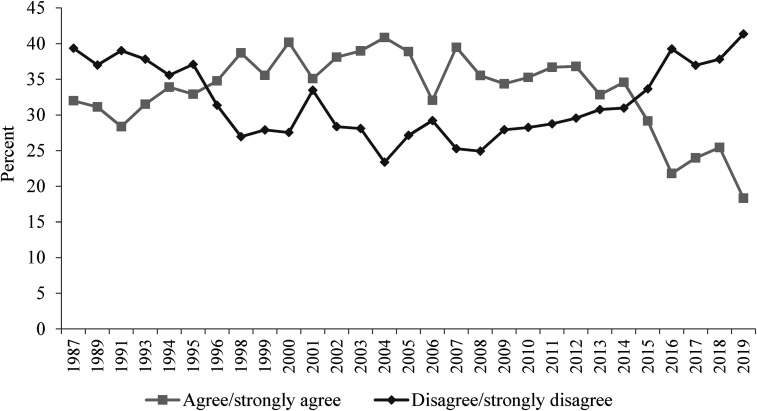
Most people on the dole are fiddling in one way or another. Source: British Social Attitudes Survey, 1987 to 2019.

There is a rather self-evident observation that the ‘anti-welfare’ orthodoxy is based on a very small number of BSA questions and Geiger and Meueleman (2016) raise further queries about the orthodoxy by examining additional BSA questions. For example, two BSA questions ask people to agree/disagree with the statements ‘Many people who get social security don’t really deserve any help’ and ‘Most people on the dole are fiddling in one way or another’. Geiger and Meueleman (ibid.) use data up to 2013 to argue that the proportion of people agreeing with these statements has barely risen since the late 1980s. In Figures 6 and 7 data are included for these questions through to 2019. Figure 6 shows that since 2013 there has in fact been a significant positive change with the proportion of BSA survey respondents saying many people don’t really deserve any help having fallen markedly from 33 per cent in 2013 to 15 per cent in 2019. In 2019 responses are far less hostile than in 1987 when the question was first asked and at which point 31 per cent of respondents agreed. A similar pattern is evident with the question about ‘fiddling’. In 1987 32 per cent of people agreed and 32.9 per cent did so in 2013, but this fell to 25 per cent in 2018.

**Figure 8.**
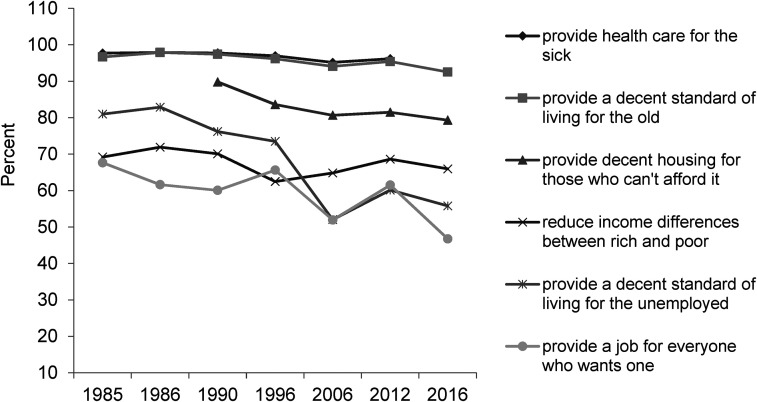
It is the government’s responsibility to… Source: British Social Attitudes Survey, 1985 to 2016.

Other BSA questions, which are often overlooked, can also be drawn on and provide evidence not only of possible doubt about the ‘anti-welfare’ orthodoxy but demonstrate the existence of ‘pro-welfare’ views. For example, the BSA regularly asks about government responsibilities. The question is posed as ‘should it be the government’s responsibility to…?’ with statements including: provide health care for the sick; provide a decent standard of living for the old; reduce income differences between rich and poor; provide decent housing for those who can't afford it; provide a decent standard of living for the unemployed; and provide a job for everyone who wants one. Data for 1985 to 2016 are presented in Figure 8 and it can be seen that:

- over 90 per cent of people from the 1980s through to 2016 say it is the government’s responsibility to provide health care for the sick and a decent standard of living for the old (respectively: 97.7 per cent in 1985, 96.2 per cent in 2012; and 96.7 per cent in 1985, 92.5 per cent in 2016 – the virtually identical top two horizontal lines in Figure 8);
- at least two thirds of people over the last thirty years consistently say it is the government’s responsibility to reduce income differences between rich and poor (69.2 per cent in 1985, 66 per cent in 2016);
- a very large majority has continued to say it is the government’s responsibility to provide decent housing for those who cannot afford it (89.8 per cent in 1990, 79.3 per cent in 2016);
- over half of people still say it is the government’s responsibility to provide a decent standard of living for the unemployed. However, support for this has fallen significantly, from 81 per cent in 1985 to 55.8 per cent in 2016, as has the percentage of people saying the government is responsible for providing a job for everyone who wants one (67 per cent in 1985 to 46.8 per cent in 2016).

Data from surveys other than the BSA also offer potential challenges to the ‘anti-welfare’ orthodoxy. For example, a highly relevant piece of work was undertaken by the Webb Memorial Trust This included qualitative work and a large-scale survey of 10,000 adults, to investigate the question ‘what kind of society do we want?’ (Knight, 2015). Pilot research identified seventeen components/qualities of a good society. The survey then asked respondents to rate the importance of each component.

**Figure 9.**
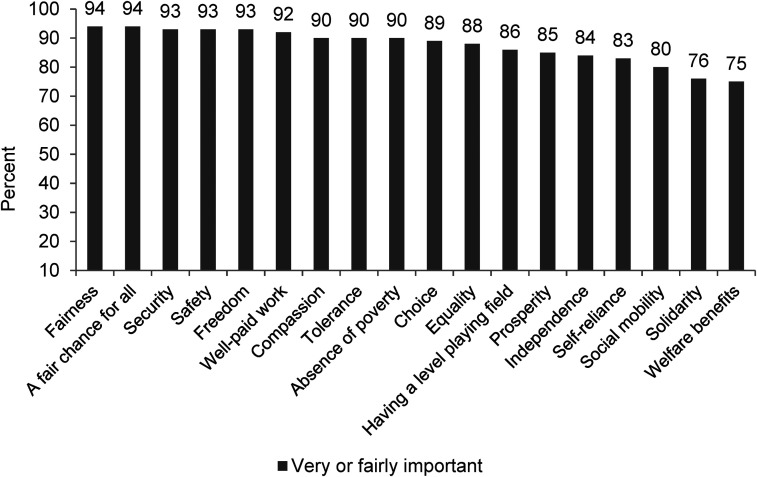
Components/qualities of a good society. Source: Adapted from Knight (2015) Chart 2, p24. Data collected by YouGov in 2015.

The results are shown in Figure 9 and are striking:

- 90 per cent of respondents said an absence of poverty is very/fairly important in a good society;
- 75 per cent said welfare benefits are a very/fairly important component of a good society; and
- large majorities also said very/fairly important qualities of a good society include compassion and tolerance (both 90 per cent), equality (88 per cent), having a level playing field (86 per cent) and solidarity (76 per cent).

If the ‘anti-welfare’ orthodoxy is as unequivocal as often claimed, then it would seem fair to expect it to be reflected in responses across BSA questions and in other surveys: but the data show that is not so. There is certainly evidence of hardening attitudes on some BSA questions and disputing that is misplaced. The key point, however, is that hardening attitudes is not the whole story. Hudson et al. (2016a) argue that ‘welfare commonsense’ has crowded out alternative narratives and understandings, but it is evident from the analysis of survey data that there are dimensions to understanding attitudes that go beyond the single ‘anti-welfare’ orthodoxy. This suggests the need for a fuller, more multi-dimensional understanding of ‘welfare attitudes’ and this can be developed further by consideration of a number of broader themes, as follows.

### (Mis)understanding ‘welfare attitudes’

This section considers four themes referred to in the introductory discussion - methodology, ‘messiness’ and ambivalence in attitudes, attitudes and constructions of deservingness, and following or leading opinion - each of which has implications for understanding attitudes and the ‘anti-welfare’ orthodoxy.

## Methodological issues

Methodology can sometimes be neglected in studies of attitudes, but Chung et al. (2018) and Taylor-Gooby et al. (2018) have raised a number of important points. The former highlight three substantial shortcomings in attitudes research: how the assumptive worlds of social scientists and policymakers shape the framing of ‘welfare attitudes’; causality and limitations in understanding how changes in policies can lead to changes in ‘welfare attitudes’; and populations covered in research. As one example, Chung et al. (2018) identify distinctions between approaches that take survey findings at face value, and those that suggest that the preconceptions of researchers shape and frame the way in which attitudes are addressed and measured, casting emphasis on some issues and downplaying or ignoring others. Also, short-comings in measurement techniques, instruments used and the samples available for analysis, potentially obscure some issues.

Chung et al. (2018) provide a detailed discussion but for the purposes of this article the point to make is that methodological concerns should at the very least prompt some caution with regard to the certainty of assertions about hardening attitudes. A more specific example relates to the far from original but still hugely important point about the importance not just of the framing of surveys, as highlighted by Chung et al. (ibid.) but the wording of individual questions. With ‘welfare attitudes’ this is illustrated by a case from Anthony Wells’ *UK Polling Report* website. In response to a government announcement in 2012 that benefits would increase by only 1 per cent, four polls were conducted to explore public attitudes to this. One poll was conducted each by YouGov and Mori and two by ComRes. The results are shown in Table 1. What is immediately apparent is that Polls 1, 2 and 3 were very consistent. When asked if benefits should increase by the rate of inflation or more, there was only a two percentage point difference (33 per cent to 35 per cent) in those agreeing. There was a little more divergence in the range of those agreeing benefits should increase by less than the rate of inflation (33 per cent to 44 per cent) but it is still a largely consistent finding across the three polls. However, Poll 4 is dramatically different. It found a significant majority (69 per cent) saying benefits should increase by the rate of inflation or more and only 16 per cent agreeing benefits should increase by less than the rate of inflation.

**Table 1.** Public attitudes to the 1 per cent increase in benefits, 2012.

	Benefits should increase by the rate of inflation or more	Benefits should increase by less than the rate of inflation
Poll 1	35%	33%
Poll 2	33%	44%
Poll 3	36%	42%
Poll 4	69%	16%

Source: Compiled from Anthony Wells’ *UK Polling Report* website - http://ukpollingreport.co.uk/.

Wells analysed why Poll 4 was so different. It could of course simply have been a rogue survey but Wells found that there was one methodological difference between the four polls. This was that the question in Poll 4 included a list of benefits while the other polls did not. The listing of benefits triggered a very different response. Wells speculates that perhaps mention of Child Benefit was the cause of people responding differently. So while three polls adhered to ‘anti-welfare’ orthodoxy, a differently worded question on the same topic produced a significantly different answer. It is evident there is need for caution in ascribing absolute certainty to ‘welfare attitudes’.

### ‘Messiness’ and ambivalence

Following on from methodological issues, the desire to present certainty about public attitudes can ignore the reality that people’s views are often complex, ambiguous and contradictory (Orton and Rowlingson, 2007: ix). There is a ‘messiness’ about people’s attitudes that should always be borne in mind. Regarding ‘welfare attitudes’, there is a particular issue about ambivalence. Geiger and Meueleman (2016: 301) argue that it is ambivalence that most characterises attitudes to the benefits system with this seeming to be true of all countries at all times – even when the benefits system is generous and popular, many people still have some concerns. The same is true for Britain today (ibid.), although in reverse i.e. even though attitudes are usually felt to be predominantly hostile, many people have positive elements to their attitudes to the benefits system.

Another indication of ambivalence can be seen in the high level of ‘Don’t knows’ in response to some of the relevant BSA questions. For example, in response to the question which asks ‘If welfare benefits weren't so generous, people would learn to stand on their own two feet’ the percentage of respondents answering ‘Neither agree nor disagree’ has, since 1987, never been below 21 per cent and stood at 27.6 per cent in the most recently available survey in 2019 (Table A1). In response to the question which asks whether ‘Government should spend more money on welfare benefits for the poor, even if it leads to higher taxes’ the percentage of respondents answering ‘Neither agree nor disagree’ has, again since 1987, never been below 22 per cent and in 2019 was 32 per cent (Table A2). Looking at these two questions, it can be seen that over the last thirty years there have always been at least one in five respondents saying they neither agree nor disagree and currently it is at least one in four people who say so.

**Figure 10.**
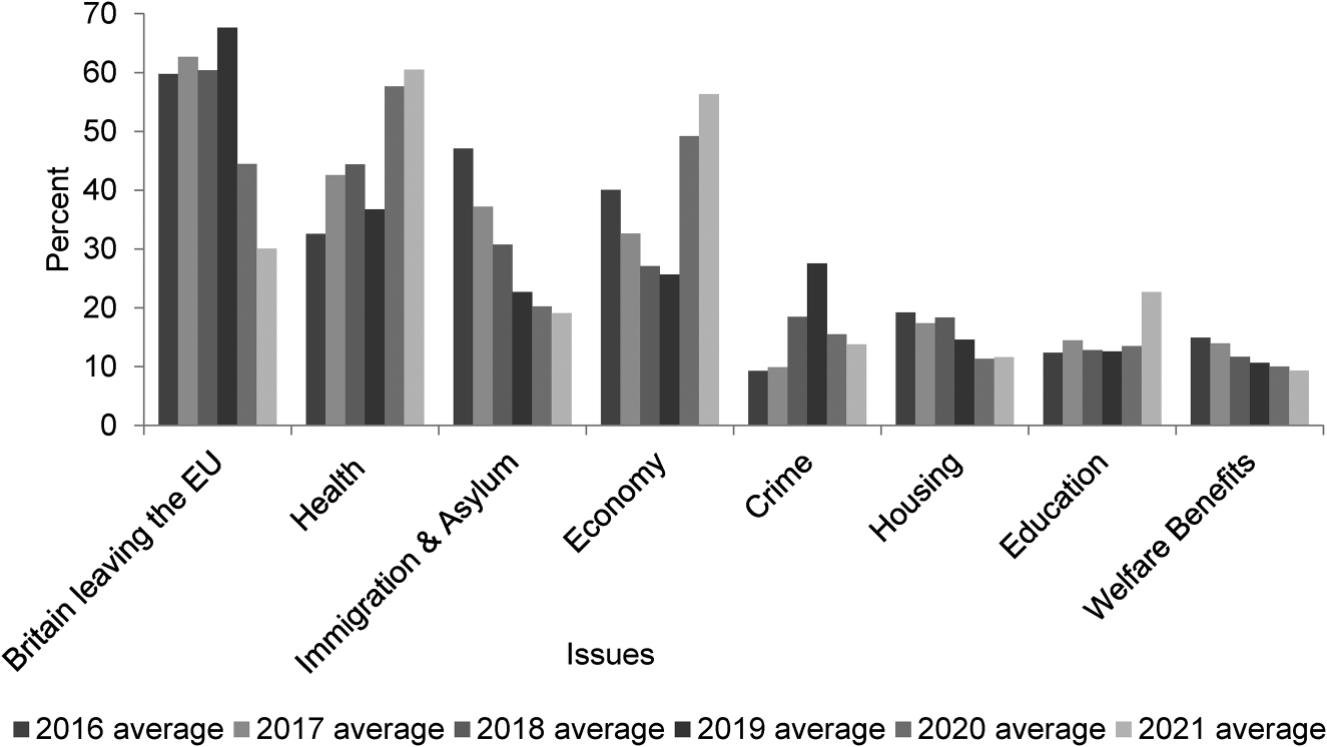
Which of the following do you think are the most important issues facing the country at this time? please tick up to three. Source: Adapted from YouGov Top issues tracker available at https://d25d2506sfb94s.cloudfront.net/cumulus_uploads/document/rxjfzj7fv0/YG%20Trackers%20-%20Top%20Issues_W.pdf. Note: The yearly averages are calculated from the available data points.

Ambivalence is also evident in people not necessarily seeing benefits as a key issue. Yougov conducts a ‘Top Issues Tracker,’ a survey which asks people to say, from a set list of issues, which three are the most important the country is facing. Data for 2016 to 2021 are presented in Figure 10.^5^ The top issue identified throughout 2016 to 2019 was Britain leaving the EU. In 2020 and 2021 the top concerns became Health and the Economy.

‘Welfare benefits’ is one option in the set list of issues. From 2016 to 2021 it has been cited as one of the top three issues facing the country by between 10 per cent and 15 per cent of respondents. Benefits can be grouped with concern about crime, education and housing, although in recent years (on average) concern with those issues has been greater than it has been for benefits. From Figure 10 it appears that people do not see benefits as critical compared with other issues.

### Attitudes and constructions of deservingness

Another issue to consider is that the survey data on which the hardening attitudes thesis is based, do not shed light on who respondents imagine the recipients of ‘welfare’ to be. The importance of this is not solely in relation to dimensions such as age or gender but more fundamentally, as Baumberg Geiger (2021) argues, that social security systems cannot be understood without considering whether claimants are perceived by the public to ‘deserve’ support. The notion of desert is therefore significant. Indeed, the issue has deep roots and can be seen in the Elizabethan Poor Laws of the late sixteenth and early seventeenth centuries, in which a central goal was to distinguish between the ‘deserving’ and the ‘undeserving’ poor in order to identify those in ‘genuine’ need of relief (Stone, 1984). The social construction of desert was cemented in the 1834 Poor Law, particularly in the operation of the workhouse, and continues today in the language of strivers versus skivers which was referred to above.

There is an extensive literature on desert and attitudes, with van Oorschot’s model of Control, Attitude, Reciprocity, Identity and Need (the CARIN model – see van Oorschot, 2000; van Oorschot et al. 2017; Bonoli, 2021) of particular importance. The CARIN model enables understanding of the logic of deservingness judgements assessed against the five criteria (e.g. Control is about whether a claimant is seen as to blame for their situation): but in practice it is observed characteristics that influence attitudes (Baumberg Geiger, 2021). Thus, another element of understanding ‘welfare attitudes’ is considering views in relation to observed characteristics, through the study of attitudes to particular reference groups. Commonly studied reference groups are the elderly and sick/disabled people, who are widely viewed as most deserving of state support, whereas unemployed people and migrants are seen as less deserving (ibid.).

As an example of unpacking this further, Baumberg Geiger (ibid.) studied perceptions of deservingness towards one specific reference group – disabled people. Baumberg Geiger found that even in relation to this single group there was a hierarchy of desert depending on specific conditions and impairments. Wheelchair users were perceived as the most deserving followed by people with schizophrenia, back pain, fibromyalgia and depression, while those with asthma were viewed as the least deserving group of disabled people.

For the purposes of this article, the point to emphasise is how debate about constructions of deservingness demonstrates further layers of complexity relevant to understanding ‘welfare attitudes’. The survey questions on which the hardening attitudes thesis rest provide no insight on the basic point of who respondents have in mind when giving answers. Nor is there evidence of whether attitudinal changes are driven by views regarding claimants generally, shifts in views towards particular reference groups or how this may relate to constructions of desert. The credence the hardening attitudes thesis gives to a small number of BSA questions ignores multiple additional factors relevant to understanding ‘welfare attitudes’.

### Following or leading opinion

A final theme to consider in this section is about following or leading opinion. One aspect of attitudes research is how social, economic, and political shifts relate to attitude change but there can also be a tendency in some studies to present changes in public attitudes, implicitly if not explicitly, as occurring in something of a vacuum: but that is far from being the case. Discussion of this point includes Sage’s (2019) analysis of Labour Party discourse on ‘welfare’ under the leadership of Jeremy Corbyn, and Hills’ (2002) identification of New Labour’s social security/‘welfare’ policies which accorded with public attitudes and those which appeared to have led opinion. Work by O’Grady (2017) is of particular interest here. O’Grady (ibid.) undertook a study of speeches on ‘welfare’ made in the House of Commons from the late 1980s to 2015. The study concludes that declining support for the benefits system has been a top-down phenomenon. O’Grady (ibid.) argues that the shift in public opinion occurred mainly in response to changes in the way that politicians have framed and discussed both the ‘welfare’ system and its users, filtered through the media.

In more detail, O’Grady contends that from the late 1980s to 2015 there was a dramatic shift in politicians’ discourse. This occurred amongst all parties but was especially notable from Labour politicians. The shift was from benefits recipients being depicted mostly as deserving and the system talked about as a highly legitimate means of poverty alleviation to users of the system being stigmatised and benefits depicted as ineffectual or even wasteful. In particular, from the late 1990s to the mid-2000s – in a very sharp break from the 1980s – Labour devoted substantially less time to talking about the benefits system and its users in positive terms than it did to problems with the system and the need for reforms. Positive mentions of ‘welfare’ plummeted and were drowned out by more negative discourse. What matters is that the shifts in rhetoric did not occur after public opinion changed. Politicians were not responding to public opinion, they were leading it.

Following or leading opinion points to a very different way of understanding attitudes. Chung et al. (2018) argue that ‘welfare attitudes’ are pivotal in understanding the preferences and demands of citizens to help shape future policy. That argument is correct if opinion is being followed. But if attempts have been made to lead opinion, then changes in attitudes can be seen as an indicator of the success or otherwise of those attempts. From this perspective, understanding ‘welfare attitudes’ is not about seeing what might be possible in relation to policy development but identifying whether attempts to lead opinion in a particular direction have succeeded. The key point, however, is that the idea of following or leading opinion challenges the very way of thinking about ‘welfare attitudes’.

The implications of the findings from our analysis will now be discussed.

## Discussion: Re-setting debate

Reflecting on the above, the overall finding can be summarised as being that ‘anti-welfare’ orthodoxy has always been far more equivocal than claimed, with the issue of following or leading opinion also of particular importance. But why, to whom and for what purposes, does it matter that public attitudes towards ‘welfare’ are not as negative as often imagined? There are a number of potential answers and several themes that could be explored further. For example, the evident equivocation provides challenges to debate about the relationship between public attitudes and policy making and attempts at building explanatory models (e.g. see Wlezien and Soroka, 2007).

The findings could also be used in relation to arguments regarding the future of neo-liberalism. Current debate includes issues such as whether neo-liberalism is now dead as a popular ideology (Cooper, 2021), moves towards national capitalism (Eaton, 2021), questioning of globalisation and a return to state intervention in the economy (Khurana and Narayan, 2021), and even the possible emergence of a new consensus (Mazzucato, 2021). Data examined in this article could certainly be used to develop a narrative that the pre-2015 years appear as something of a high water mark in efforts by governments and media to harden attitudes to ‘welfare’, but that limits were reached. Similarly, the softening of attitudes since 2015 could potentially reflect a turn against the politics and policy of austerity, and be a possible indication of broader dissatisfaction with neo-liberalism.

But caution is required. A theme of this article has been the limitations of the survey questions on which the hardening attitudes thesis rests. To infer, from responses to that narrow set of questions, findings regarding broader issues of political economy is not necessarily plausible. Further caution is also required as claims about the death of neo-liberalism must themselves be treated warily given the ideology’s adaptiveness (Peck and Tickell, 2002; Peck and Theodore, 2019), its ability to withstand and adjust to shocks, such as the 2008 global financial crisis (Crouch, 2011) and variegation in the neo-liberal project across different policy areas, national settings and time periods (Humpage, 2015). The data we have discussed may act as a piece in a larger jigsaw, certainly demonstrating a limit in the success of entrenching a neo-liberal view of ‘welfare’ and even growing rejection of that view, but to reach robust conclusions about neo-liberalism requires further research evidence.

Where the findings have more particular and immediate relevance is in relation to poverty and its redress. It is from anti-poverty actors that much of the concern with the hardening attitudes thesis has arisen. Baumberg Geiger and Meueleman (2016) note in relation to anti-poverty action that the dominant view has become that the public do not have an accurate view of the benefits system, instead believing myths and holding negative attitudes. A central tactic of many think tanks and campaigning organisations has consequently been to use mythbusting approaches which attempt to shift public attitudes through evidence based challenges to flawed beliefs (Hudson et al., 2016a). Action is guided by a view that social security can only be rebuilt when negative attitudes are tackled (Baumberg Geiger and Meueleman, 2016).

The finding that the hardening attitudes orthodoxy is not as unequivocal as claimed is therefore a positive one for anti-poverty actors, and there is some evidence of this being recognised in terms of efforts to re-set debate. For example, the Joseph Rowntree Foundation (2018) published the outcome of a two year project on how to build lasting support to solve UK poverty. This rejects mythbusting and instead argues for what is described as developing a new narrative explaining why tackling poverty matters and framing this in terms of shared values of compassion and justice. Such an approach can be seen as according with what Baumberg Geiger and Meueleman (2016) describe as the need to emphasise the positive rather than negative side of the ambivalence in ‘welfare attitudes’.

A fuller understanding of attitudes and seeking to connect with positive rather than negative elements of ambiguity are important steps but in terms of a more fundamental re-setting of debate there is a further reflection that can be made, relating to the finding about the importance of following or leading opinion. Anti-poverty actors may want the public to have less negative attitudes to ‘welfare’ but to re-set debate demands something more than this. In short, re-setting debate means leading not following opinion and to do that requires a clearly articulated, credible alternative to ‘welfare reform’. But as has been argued elsewhere (Batty and Orton, 2018) there is currently a lack of consensus among progressives as to what that alternative might be. There is no shortage of ideas (ibid.) but there is a lack of agreement for example on whether Universal Credit should be reformed or abolished, the role of contributory National Insurance benefits, the balance between universalism and means-testing plus the highly polarised debate about the concept of a universal basic income.

The consequence of there not being a cogent progressive alternative to ‘welfare reform’ is evident in some of the studies of attitudes discussed above. For example, O’Grady (2017) notes that with ‘welfare’ depicted as ineffectual and wasteful and claimants stigmatised the public is simply unused to hearing an alternative in which ‘welfare’ and ‘welfare recipients’ are talked about in positive terms. Taylor-Gooby et al. (2018: 922) found in their deliberative forums on ‘welfare’ that there were positive views including commitments to fairness and meeting the needs of vulnerable groups, for example by scrapping zero–hours contracts and maintaining provision in health care and pensions. But participants were unable to offer a framing that brought the positive ideas together to form an effective counter to the much stronger framing of ideas that linked unsustainable spending and misguided government with the value of individual responsibility. Stronger (negative) views therefore emerged as a consensus and weaker (positive) views became lost

So the final point to make is that for anti-poverty actors, perhaps somewhat counter-intuitively, effort needs to focus not so much on analysis of current attitudes but setting out an alternative to the ‘welfare reform’ agenda. That is the means to being able to lead rather than follow opinion and re-set debate in a new, progressive direction.

## Conclusion

The pandemic has witnessed emerging debate about a possible shift in ‘anti-welfare commonsense’ but seeing Cov-19 in itself as the cause of a shift from hardening to more positive views is to misunderstand attitudes on ‘welfare’. Analysis of survey data shows that attitudes on many points were softening pre-pandemic, in some cases to the extent that contemporary attitudes are less hostile than in the 1980s/90s. There is even evidence of ‘pro-welfare’ attitudes – completely antithetical to the ‘anti-welfare’ orthodoxy. Consideration of broader issues relating to analysis of attitudes - methodology, ‘messiness’, ambivalence, attitudes and constructions of deservingness and following or leading opinion - all point to the need for a fuller, more multi-dimensional understanding of ‘welfare attitudes’. The key finding is that ‘anti-welfare’ orthodoxy has always been far more equivocal than claimed, with the issue of following or leading opinion also of particular importance.

There are implications for a number of debates, including the relationship between public attitudes and policy making and the future of neo-liberalism as a popular ideology, but concern with the hardening attitudes thesis has primarily come from anti-poverty actors and this is where the findings have particular relevance. A positive point for those seeking the redress of poverty is public attitudes on ‘welfare’ are not as negative as often assumed. The challenge is that to re-set debate requires leading not following opinion and that in turns means having a credible alternative to ‘welfare reform’. But there is currently a lack of consensus on what the alternative should be so the conclusion is that to re-set debate is not so much about further analysis of attitudes but articulating a cogent progressive position on social security.

## Notes

1. From the outset a point needs to be made regarding language. Following the example of Hudson et al. (2016a: 215–216), it is noted that the growing usage of the term ‘welfare’ has become part of pejorative political discourse, its usage implying social assistance for the poor, tied to the notion of ‘dependency’ and (quoting Lister, 2011): 'This American import has not only besmirched the concept of welfare, but also displaced the term “social security”'. With ‘welfare’ problematised, ‘welfare reform’ becomes the dominant agenda (in practice, a harsher, more punitive system which fails even to prevent destitution). As Hudson et al. (2016a) argue, ‘welfare’ may have become the popular descriptor for social protection, mainstreamed in its usage by politicians, the media and, increasingly, academics, but it is a very loaded term. To reflect this, Hudson et al. (ibid.) adopt what they describe as ‘the somewhat unwieldly convention’ of placing ‘welfare’ in inverted commas. This article follows that convention. Language is also important more broadly. For proponents of ‘welfare reform’, ‘anti-welfare commonsense’ is a positive not a negative. Terms used in this article, such as ‘hardening’, ‘softening’ and so on, are not value-neutral.

2. *Critical Social Policy* has also published a themed issue on the topic of policies and discourses of poverty during a time of recession and austerity (see Pantazis, 2016) which while not directly relevant to the examination of public attitudes in this article is of related broader interest.

3. Sources in this paragraph are: www.opendemocracy.net/en/opendemocracyuk/pandemic-poverty-i-cant-afford-to-live-on-94-a-week/www.bbc.co.uk/news/business-52005581; www.telegraph.co.uk/women/politics/day-marcus-rashford-kicked-boris-johnson-action/ (accessed 19 January 2021).

4. BSA data used in this article are available from the UK Data Service: https://beta.ukdataservice.ac.uk/datacatalogue/series/series?id=200006#!/abstract*.*

5. Source: https://d25d2506sfb94s.cloudfront.net/cumulus_uploads/document/yvvp1agvnp/YG%20Trackers%20-%20Top%20Issues_W.pdf (accessed 8 August 2018).

## Appendix

**Table A1.** If welfare benefits weren't so generous, people would learn to stand on their own two feet.

YEAR	Agree strongly	Agree	Neither agree nor disagree	Disagree	Disagree strongly	Not answered	Total
1987	7.8	25.1	21.3	31.6	13.7	0.5	100
1989	7.6	22.6	23.1	33.4	12.3	0.8	100
1991	6.4	19.3	22.7	36.8	13.5	1.3	100
1993	5.6	19.7	22.0	36.1	15.6	1.0	100
1994	5.4	21.3	22.6	36.5	12.5	1.8	100
1995	7.7	25.3	21.3	32.8	10.8	2.1	100
1996	8.3	25.0	23.5	29.7	11.9	1.6	100
1998	9.3	30.3	26.3	27.4	4.7	1.9	100
1999	7.5	30.2	26.7	28.9	4.9	1.9	100
2000	7.3	30.9	25.3	30.8	4.4	1.3	100
2001	8.6	30.6	23.7	30.2	5.3	1.6	100
2002	9.9	33.9	23.9	25.3	4.6	2.3	100
2003	11.4	30.2	27.3	25.3	3.3	2.6	100
2004	11.4	35.4	27.3	20.1	3.5	2.4	100
2005	12.1	37.4	24.0	21.5	3.1	2.0	100
2006	8.8	37.8	26.1	22.8	2.2	2.1	100
2007	13.0	39.5	22.5	19.5	2.8	2.7	100
2008	12.7	40.8	23.8	17.5	2.7	2.5	100
2009	11.1	41.5	23.5	19.7	2.6	1.7	100
2010	12.5	42.7	23.1	15.8	3.9	2.0	100
2011	14.7	39.2	23.2	17.9	3.3	1.7	100
2012	14.3	38.3	23.2	17.5	4.3	2.4	100
2013	14.5	38.4	22.0	18.7	3.8	2.6	100
2014	13.6	39.3	23.1	17.4	5.2	1.6	100
2015	12.2	39.8	22.3	19.3	4.7	1.7	100
2016	9.7	33.6	25.5	23.4	5.9	2.0	100
2017	9.7	33.4	25.0	22.9	6.8	2.1	100
2018	8.2	31.3	26.7	24.1	8.3	1.6	100
2019	7.8	25.6	27.6	27.1	9.9	2.0	100

Source: British Social Attitudes Survey, 1983 to 2019.

**Table A2.** Government should spend more money on welfare benefits for the poor, even if it leads to higher taxes.

YEAR	Agree strongly	Agree	Neither agree nor disagree	Disagree	Disagree strongly	Not answered	Total
1987	16.3	38.3	22.8	19.3	2.6	0.7	100
1989	18.3	42.3	23.0	13.9	1.5	1.0	100
1991	15.2	42.9	23.1	15.7	1.8	1.4	100
1993	14.0	38.9	25.4	17.5	2.6	1.5	100
1994	10.1	39.6	25.3	21.1	2.0	1.9	100
1995	10.6	38.9	25.4	20.6	2.3	2.2	100
1996	10.2	33.0	28.5	23.1	3.3	1.8	100
1998	6.9	36.0	29.2	22.9	2.9	2.2	100
1999	6.2	33.8	29.9	25.1	3.2	1.8	100
2000	6.6	31.7	31.1	26.6	3.0	0.9	100
2001	6.5	36.8	29.6	22.9	2.7	1.5	100
2002	6.7	37.2	27.2	23.3	3.2	2.5	100
2003	6.4	36.9	28.8	23.7	2.1	2.2	100
2004	4.5	31.6	29.6	28.1	3.6	2.6	100
2005	4.2	31.6	31.5	27.1	3.5	2.1	100
2006	4.3	30.8	34.0	26.6	2.6	1.8	100
2007	3.8	27.7	32.7	28.9	4.1	2.8	100
2008	5.0	29.9	28.0	29.4	5.3	2.4	100
2009	3.1	23.7	28.4	38.2	4.6	2.2	100
2010	4.2	24.9	30.5	32.4	6.2	1.9	100
2011	4.2	23.6	31.8	33.3	5.3	1.8	100
2012	5.7	28.2	32.3	26.4	5.1	2.3	100
2013	5.7	30.3	29.5	26.4	5.3	2.8	100
2014	4.5	25.4	29.8	32.5	6.3	1.4	100
2015	6.1	32.7	28.6	26.7	4.1	1.8	100
2016	5.8	29.0	33.5	26.1	3.5	2.0	100
2017	7.6	35.9	28.1	23.2	3.6	1.6	100
2018	6.8	34.5	30.5	23.5	3.3	1.4	100
2019	8.5	33.5	32.2	20.9	3.1	1.9	100

Source: British Social Attitudes Survey, 1983 to 2019.
